# Highly Variable Expression of *ESR1* Splice Variants in Human Liver: Implication in the Liver Gene Expression Regulation and Inter-Person Variability in Drug Metabolism and Liver Related Diseases

**Published:** 2019-09-30

**Authors:** JW Sun, JM Collins, D Ling, D Wang

**Affiliations:** 1 Department of Biological Chemistry and Pharmacology, College of Medicine, The Ohio State University, Columbus, Ohio, USA; 2 Department of Pharmacotherapy and Translational Research, Center for Pharmacogenomics, College of Pharmacy, University of Florida, Gainesville, Florida, USA

**Keywords:** (*ESR1*), Alternative splicing, Liver, Gene expression, Inter-person variability

## Abstract

Estrogen receptor alpha (*ESR1*) plays an important role in many tissues including the liver. Numerous alternative splice variants of *ESR1* exist that encode *ESR1* proteins with varying functions. We aim to study *ESR1* genomic organization and its mRNA expression profile in human liver by incorporating information from literature and genomic databases (Ensembl, NCBI and GTEx), and employing a quantitative method to measure all known *ESR1* mRNA splice variants in 36 human livers. We re-constructed *ESR1* genomic organization map that contains 29 exons. *ESR1* mRNA splice variants with varying 5’ untranslated region (5’UTR) and/or missing each of eight coding exons are readily detectable in liver and other tissues. Moreover, we found extensive inter-individual variability in splice variant pattern of *ESR1* transcripts. Specifically, *ESR1* transcripts lacking first coding exon are the main transcripts in liver, which encode *ESR1* proteins missing N-terminal 173 amino acids (for example, ERα46), reported previously to have either constitutive activity or dominant negative effects depending on cellular context. Moreover, some livers predominantly express *ESR1* transcripts missing exon 10 or 16, encoding C-terminal truncated *ESR1* proteins with varying *ESR1* activities. Inter-person variability in *ESR1* expression profile may contribute to inter-person variability in drug metabolism and susceptibility to liver related diseases.

## Introduction

Estrogen plays an important role in both reproductive and non-reproductive tissues. Two estrogen receptors have been described so far, estrogen receptor alpha (*ESR1*) and estrogen receptor beta (ESR2), both belong to the nuclear receptor superfamily. *ESR1* is known to act as ligand-induced transcription factor, however, ligand-free activity of *ESR1* has also been reported recently [1,2]. Like other members in the nuclear receptor family, *ESR1* protein consist of several functional distinct domains formed by different exons: N-terminal ligand independent transactivation domain (activation function-1, AF-1), a DNA binding domain, a hinge domain, and a ligand-binding and C-terminal transactivation domain (AF-2) [3].

*ESR1* gene is located at chromosome 6q25 locus, spanning 140 kb. Soon after it was cloned in 1986, *ESR1* was described to have only eight exons, all of which are protein coding [4]. Since then, additional exons and alternative splice variants of *ESR1* have been identified. Like other steroid hormone receptors that contain multiple promoters, *ESR1* transcription can be initiated from at least seven promoters, each with unique 5’-untranslated regions (5’UTR) [5–7]. Additionally, alternative splicing of internal exons generates numerous splice variants with either distinct 5’UTRs or encoding *ESR1* proteins missing different functional domains [8–12]. Some of these splice variants have either constitutive activity, no activity, or dominant negative activity [13–15]. Unfortunately, different terminology and exon numbering system have been used by individual researchers. This has resulted in confusion regarding different promoters or exons used in *ESR1* expression.

The liver is one of the main target tissues for *ESR1* with relatively high mRNA expression level and liver specific promoter [7]. Genetic variation in *ESR1* gene has been associated with liver related traits, for example, type 2 diabetes, coronary artery disease (CAD) [16,17].

Compared to reproductive organs, expression profile and function of *ESR1* in the liver are not well studied. Regression analysis using microarray gene expression and cytochrome P450s activity data indicates a correlation between expression of *ESR1* mRNA level and enzyme activity of several cytochrome P450s, for example, CYP3A4, CYP2C9, CYP2B6, etc. [18]. Moreover, using computational modelling and molecular genetic studies, we have recently identified ligand-free *ESR1* as a master regulator for the expression of CYP3A4 and other https://doi.org/10.1124/mol.119.116897 cytochrome P450s in human liver (Molecular Pharmacology, in press). This raises the possibility that different *ESR1* expression profiles may contribute to inter-person variability in the expression of P450 enzymes and drug metabolism.

In this study, we searched literature and genomic databases including Ensembl, NCBI and GTEx to re-construct *ESR1* genomic organization map and unify the terminology and exon numbering. We compared expression levels of *ESR1* total mRNA and splice variants in different tissues/cells using RNAseq data from GTEx. Moreover, we measured different promoter usage or exons/splice variants in liver and other tissues and evaluated the inter-person variability in the expression of *ESR1* splice variants in human livers. The results show diverse expression profiles of *ESR1* in different tissues and highly inter-person variability of *ESR1* expression profile in human livers.

## Materials and Methods

### Tissue samples

Thirty-six livers were obtained from the Cooperative Human Tissue Network (CHTN) (Supplementary Table 1). Total RNAs were prepared from these tissues as described previously [19]. Pooled total RNAs from normal breast, lung, heart, brain, and small intestine were obtained from Cell Applications (San Diego, California, USA). Complementary DNA (cDNA) was generated from 0.5 μg total RNA using oligo-dT, as well as, several *ESR1* gene-specific primers that target different exons to enhance cDNA yield and bypass partial degradation that may have occurred postmortem [20] (Supplementary Table 2).

### Quantitative analysis of splice variants

To estimate promoter usage, we used real-time PCR with specific primers to quantitate the relative expression levels of different first exons. For alternative splicing of internal exons, we used PCR amplification of cDNA using fluorescently labeled primers for splice variants analysis as we have described previously [20]. For each splice locus, a pair of PCR primers flanking the splicing site was designed using Primer Express Program (Applied Biosystem, Foster City, California, USA), with one primer labeled with fluorescent dye FAM (Table S2). After initial denaturing at 95°C for 5 min, the PCR reactions were run for 30 cycles under the following conditions: 95°C for 30 s, 60°C for 1 min and 72°C for 1 min. Then the PCR amplification products were separated in a SeqStudio (ThermoFisher, California, USA). Data are analyzed using Gene Mapper 5.0 software. Splice variants with different molecular weight yielded peaks with different retention times. The peak area for each splice variant is proportional to the amount of cDNA amplified as reported previously [20]. The minimum size difference clearly separable is 2 base pair (bp) for PCR products ranging from 100 to 1000 bp. Splice variants observed in each locus were confirmed by at least two sets of primers that gave rise to different sized PCR products. The optimal primer sets were selected for quantitative analysis (Supplementary Table 2).

### Detection of *ESR1* protein using capillary western blotting

Human liver samples were homogenized with 300 μl lysis buffer containing 10 mM HEPES pH 7.9, 137 mM NaCl, 10% glycerol, 1% NP-40, 1mM PMSF supplemented with protease inhibitor cocktail (Roche, South San Francisco, CA, USA). Total protein concentrations were measured using Bradford method (Thermofisher Scientific, California, USA). MCF7 whole cell lysates prepared with RIPA lysis buffer (Millipore Sigma) were used as a positive control. Capillary Western blot analyses were performed using the Protein Simple Jess system (Biotechne, California, USA) according to manufacturer’s protocol. Briefly, tissue or cell lysates were diluted with 0.1 × sample buffer to concentration of 1 mg/ml. Then 4 parts of diluted samples were combined with 1 part 5 × Fluorescent Master Mix (containing 5 × sample buffer, 5 × fluorescent standard and 200 mM DTT) and heated at 95°C for 5 min. Then the denatured samples, blocking reagent, mouse anti- *ESR1* antibodies (D-12 and F-10, at 1:10 dilution, Santa Cruz, California USA), HRP-conjugated anti-mouse secondary antibody (1:20) and chemiluminescent substrate (Biotechne, California, USA) were dispensed into designated wells in an assay plate. A biotinylated ladder provided molecular weight standard for each assay. After plate loading, the separation, electrophoresis and immunodetection steps take place in the fully automated capillary system.

### Databases

*ESR1* genomic structure and splice variants were collected from Ensembl genome browser 96 (https://useast.ensembl.org/index.html), National Center for Biotechnology Information (NCBI, https://www.ncbi.nlm.nih.gov/) and Genotype-Tissue Expression – GTEx Portal (https://gtexportal.org/home/). RNAseq data of *ESR1* splice variants were from GTEx (Analysis Release V6p).

### Data analysis

Data are expressed as mean ± SD. Statistical analysis was performed using Prism (GraphPad Software, San Diego, CA, USA). University of Florida Biosafety Committee and IBR Committee approved the human tissue study.

## Results

### Genomic organizaion of *ESR1*

**Figure 1:**
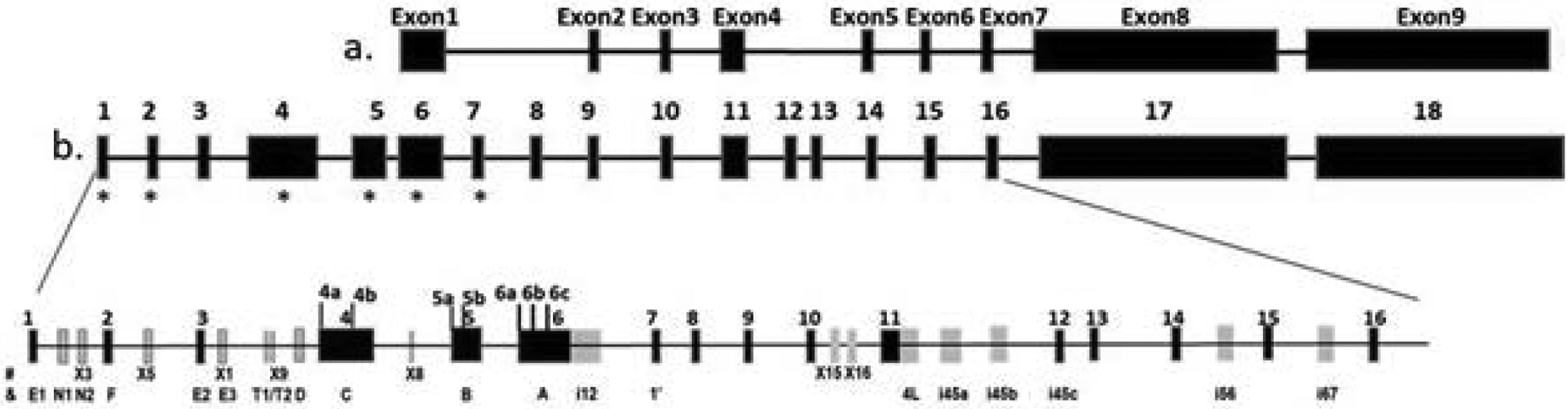
Schematic diagram of different versions of genomic organization of ESR1. (a) Traditional version of ESR1 genomic organization; (b) Upper panel, ESR1 genomic organization based on Genotype-Tissue Expression (GTEx) project; lower panel, new version of ESR1. Used as initiating exon; #exon names from NCBI database; & exon names from the literatures [7,11,12,13,22,23].

Early version of *ESR1* genomic organization contains eight or nine exons (Figure 1a), all of which are protein coding. In spite of numerous new exons have been continuously discovered, the exon numbering of this old version is still being used [13]. The most recent version of genomic organization of ERS1 contains 18 exons reported by GTEx (Genotype Tissue Expression project) (also see GTEx portal at https://gtexportal.org/home/) [21]. All of these 18 exons were detectable using RNAseq in at least one GTEx tissue sample (Fig 1b upper panel). The previous exon 1 to 9 correspond to exons 6, 9, 10, 11, 14, 15, 16, 17 and 18 of new exon numbering system. Exon 18 (or exon 9 in old version) is an alternative terminal exon used by some splice variants, for example, *ESR1*-206 (ER-α36) [22].

Searching the literature [7,11,12,14,23], Ensembl (https://useast.ensembl.org/index.html), and NCBI (https://www.ncbi.nlm.nih.gov/) databases, we found additional *ESR1* exons that are not in GTEx. Thus, we incorporated these additional exons and generated the newest version of *ESR1* genomic organization map that contains 29 distinct exons (Figure 1b). Some of them are alternative exons or exons with multiple splicing acceptor/donor sites, for example exons 4, 5, 6 and 11, leading to numerous splice variants of *ESR1*.

### *ESR1* transcripts or splice variants reported in GTEx and NCBI database or in the literature

Fifteen transcripts are in Ensembl database. Thirteen of them (from *ESR1*-201 to *ESR1*-213) are also in GTEx (Supplementary Table 3a). Twenty-one transcripts are in NCBI database (Table S3b), two of them are overlap with Ensembl database (variant 2 and 4), while others are splice variants or contain exons that have not been reported previously (denoted as EX1, EX3, EX5, etc) (Supplementary Table 3b). The structures of these transcripts are shown in (Supplementary Figure 1).

Transcription of *ESR1* is known to start from multiple promoters and different first exons, generating A, B, C, D, E, F isoforms, as well as a testis specific T isoform [7,13,14,23]. Previously denoted exons 1A, 1B, 1C, 1E, and 1F correspond to exon 6, 5, 4, 2, and 1 of new numbering system (Figure 1b). With another first exon, exon 1’ (exon 7 in new numbering system), reported by Wang et al. transcription of *ESR1* can be initiated from at least 6 different exons, labeled with a star, in non-testis tissues (Figure 1b) [22]. Moreover, alternative usage of several non-coding upstream exons generates numerous 5’UTR splice variants of transcripts initiated from exon 1 or 2 (Supplementary Table 3c) [11,12,23].

In addition to alternative splicing of 5’UTR, alternative splicing of internal exons generates splice variants lacking different coding exons, resulting in *ESR1* protein lacking functional domains. Transcripts lacking each of eight coding exons, singly or in combinations, have been reported in tissues or cells from normal or disease conditions [8–10] (Supplementary Table 3d). Moreover, a recent study demonstrates a spectrum of C-terminal truncated *ESR1* protein generated from the usage of several newly identified intronic exons (for example, i45a, i45b, i45c, i56 and i67, (Figure1b) (Supplementary Table 3) [13].

Adding to the diversity, some *ESR1* transcripts, for example, ERα36 or *ESR1*-206 or variant X11 use alternative terminal exon, exon 18, encoding an (*ESR1* protein with distinct C-terminal domains (Supplementary Figure 1b) [22]. Furthermore, usage of different polyadenylation sites generates *ESR1* variants with different lengths of 3’UTR (long or short, L or S), potentially subject to different regulations by miRNAs [24] (Supplementary Figures 1a and 1b).

### Expression of *ESR1* transcripts in different tissues/cells

#### RNAseq data from GTEx:

**Figure 2:**
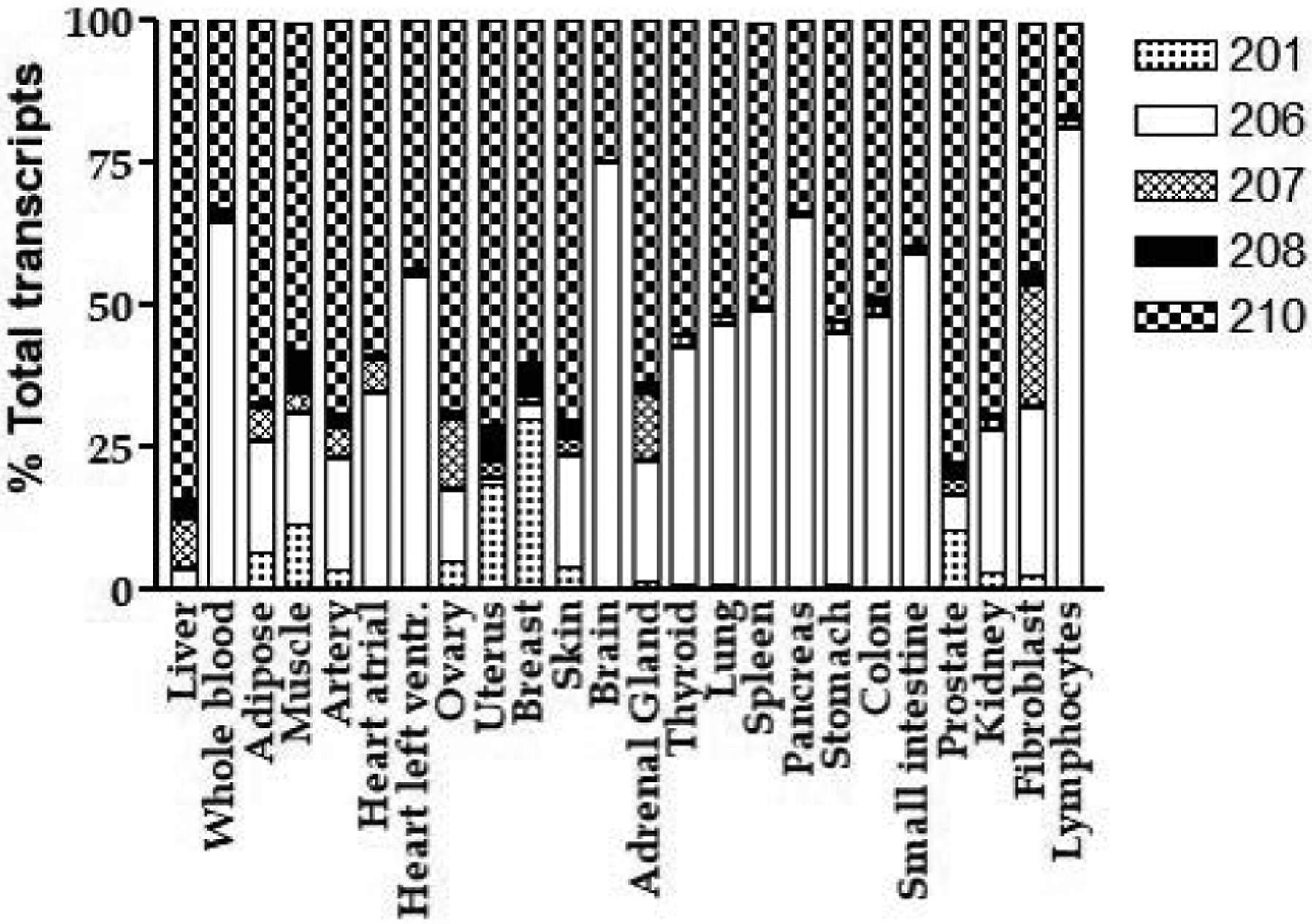
Relative expression levels of different transcripts in 24 human tissues measured using RNAseq. Data from GTEx project.

Total *ESR1* RNA expression levels in different tissues/cells or brain regions from RNAseq (GTEx data) are shown in Figure S2. The expression levels of *ESR1* vary drastically in different tissues, with highest expression levels in reproductive tissues and pituitary, followed by liver, and lowest in brain. At transcript level, the expression of eight *ESR1* transcripts (*ESR1*-202, 203, 204, 205, 209, 211, 212 and 213 (Supplementary Figures 1a and 1b for structure of these transcripts) are low, only detectable in a small fraction of samples of a given tissue, thus, these transcripts are excluded from further analysis. Five *ESR1* transcripts (*ESR1*-201, 206, 207, 208, 210), driven by different promoters upstream of exons 6, 7, 2, 5, and 4, respectively, were expressed in the majority of samples of a given GTEx tissue. Figure 2 shows tissue specific expression pattern, with *ESR1*-210 or *ESR1*-206, driven by promoter upstream of exons 4 and 7, being the main transcripts in most of the tissues, including reproductive tissues, breast, ovary, and uterus. GTEx data show the main transcript in liver is *ESR1*-210 that initiated from exon 4 and with an exon 11 deletion (Figure 2, Supplementary Figure 1a).

#### Real-time PCR analysis:

**Figure 3:**
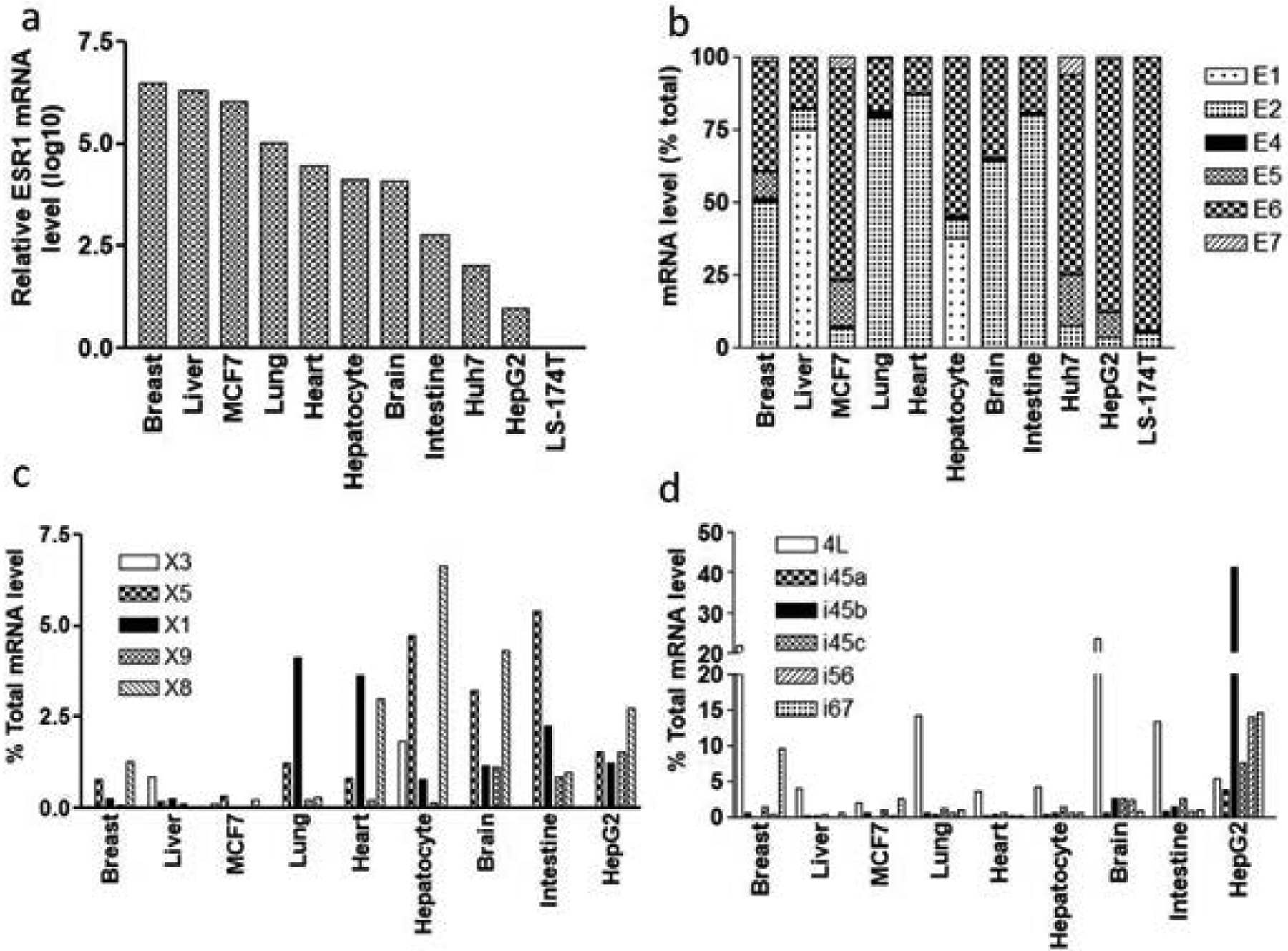
ESR1 expression profiles in different tissues/cells as indicated measured with real-time PCR. (a) Total ESR expression; (b) Relative expression levels of different first exons; (c) Expression of unknown exons of ESR1; (d) Expression of intronic exons of ESR1.

To validate RNAseq results from GTEx, we measured the expression of total *ESR1* RNA in 11 tissues/cells using quantitative real-time PCR. Shown in Figure 3a, liver expresses relative high level of *ESR1*, next to that in breast, consistent with RNAseq results from GTEx (Supplementary Figure 2).

We then measured the expression levels of six starting exons, exon 1, exon 2, exon 4, exon 5, exon 6, and exon 7, in these 11 tissues to estimate the usage of different promoters. Shown in Figure 3b, in most of the tissues tested, exon 2 or exon 6 are the most highly expressed exons, consistent with broad expression patterns of these two exons [7]. Exon 1 is only expresses in liver and primary hepatocytes, consistent with previous study suggesting liver specific promoter upstream of exon 1 [7,11]. However, measured first exon (or promoter) usage does not agree with RNAseq data from GTEx, in which exon 7 or exon 4 are the main starting exons (*ESR1*-206 and *ESR1*-210, Figure 2) for most of the tissues tested including liver.

We then tested the expression of exons not in GTEx but reported in NCBI database or in the literature (exon in grey color), including unknown exons EXs and newly identified intronic exons in different tissues/cells [13] (Figure 1b, Supplementary Figure 1b). Shown in Figures 3c and 3d, except for exon X1, which is undetectable in lung, heart, brain, intestine, and HepG2 cells, all other exons are detectable in tissues/cells tested. However, most of these exons express at low levels (less than 10% of total expression levels), except for exon 4L and i45b (Figure 3d). Exon 4L (intron 11 retention) expresses relatively high level in breast, lung, and brain but low in liver, while exon i45b expresses at high level in HepG2 cells (Figure 1b). Variants with intron 11 retention or containing i45b encode C-terminal truncated *ESR1* proteins [25]. This result validates expression of these newly identified exons in normal human tissues.

### Expression of *ESR1* splice variants in human livers

We used PCR with fluorescently labeled primers followed by capillary electrophoresis as we have reported previously, to screen for different splice variants of *ESR1* in human livers [20]. PCR primers were designed to capture the expression of transcripts driven by three different promoters with three different first exons (exon 1, 2 and 6), and to cover the entire *ESR1* coding region. Six loci were analyzed: exon 1 to exon 9 (E1-E9); exon 2 to exon 10 (E2-E10); exon 6 to exon 10 (E6-E10); exon 9 to exon 15 (E9-E15); exon 10 to exon 15 (E10-E15); and exon 11 to exon 17 (E11-E17). Of 13 known splice variants initiating from exon 1 and 2, 10 of them are clearly detectable in the liver, including one with exon N2 or X3 insertion, not reported in GTEx (Supplementary Table 3c, Supplementary Figure 3, Figure 1b). Moreover, alternative splicing of transcripts initiated from exon1 and 2 also generate splice variants lacking first coding exon, exon 6 (Supplementary Figure 3a). Furthermore, *ESR1* transcripts missing each of other 6 coding exons (Δ9, Δ10, Δ11, Δ14, Δ15 or Δ16), singly or in combinations, are detectable in human livers, as were reported in other tissues/cells [8–10] (Supplementary Figure 4). Deletion of last exon cannot be determined because it is used to design reverse PCR primer.

### Inter-person variability in the expression of *ESR1* splice variants in human livers

**Figure 4:**
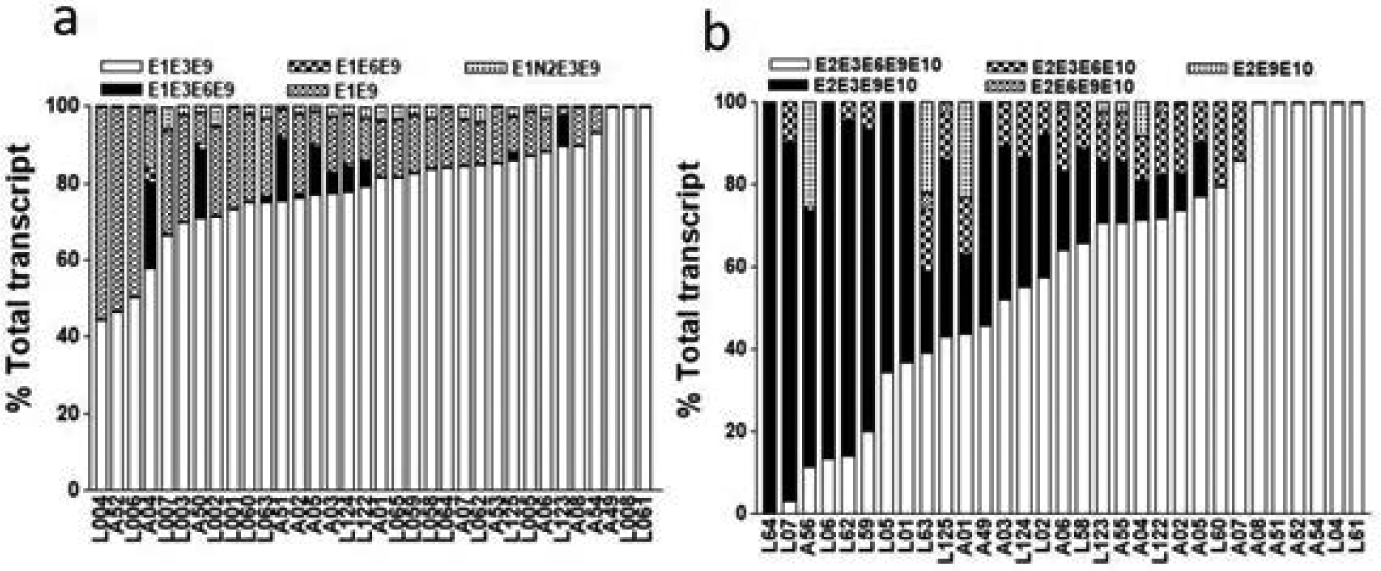
The patterns of ESR1 splice variants initiated from exon 1 (exon 1 to exon 9) (a) or exon 2 (exon 2 to exon 10) (b). The amount of each splice variant was expressed as the percentage of the total transcripts from each locus. Each vertical bar represents the composition of ESR1 splice variants in human liver from different individuals. Each sample was measured twice, and mean is shown.

We then quantitated the relative expression levels of each splice variant of *ESR1* in 36 human livers (see Supplementary Table 1 for donor demographics). For transcripts initiating from exon 1, the most abundant splice variant is E1-E3-E9, followed by E1-E9, both of them lack exon 6 (Figure 4a). Only a small portion of liver samples tested show significant portion of exon 6 containing variant (E1-E3-E6-E9 or E1-E6-E9) (Table 1 and Figure 4a). In contrast, for transcript initiated from exon 2, both exon 6 containing (e.g. E2-E3-E6-E9-E10), and exon 6 skipping (eg. E2-E3-E9-E10) variants are variably expressed in different livers, with the proportion of both variants ranging from 0% to 100% of total transcripts in different livers (Table 1 and Figure 4b). Other variants missing exon 3, 9, or both are sporadically expressed in some livers. The results indicate that exon 6 skipping transcript that encodes an N-terminal truncated *ESR1* protein, for example, ERα46, is a predominant *ESR1* isoform in human livers [14].

**Figure 5:**
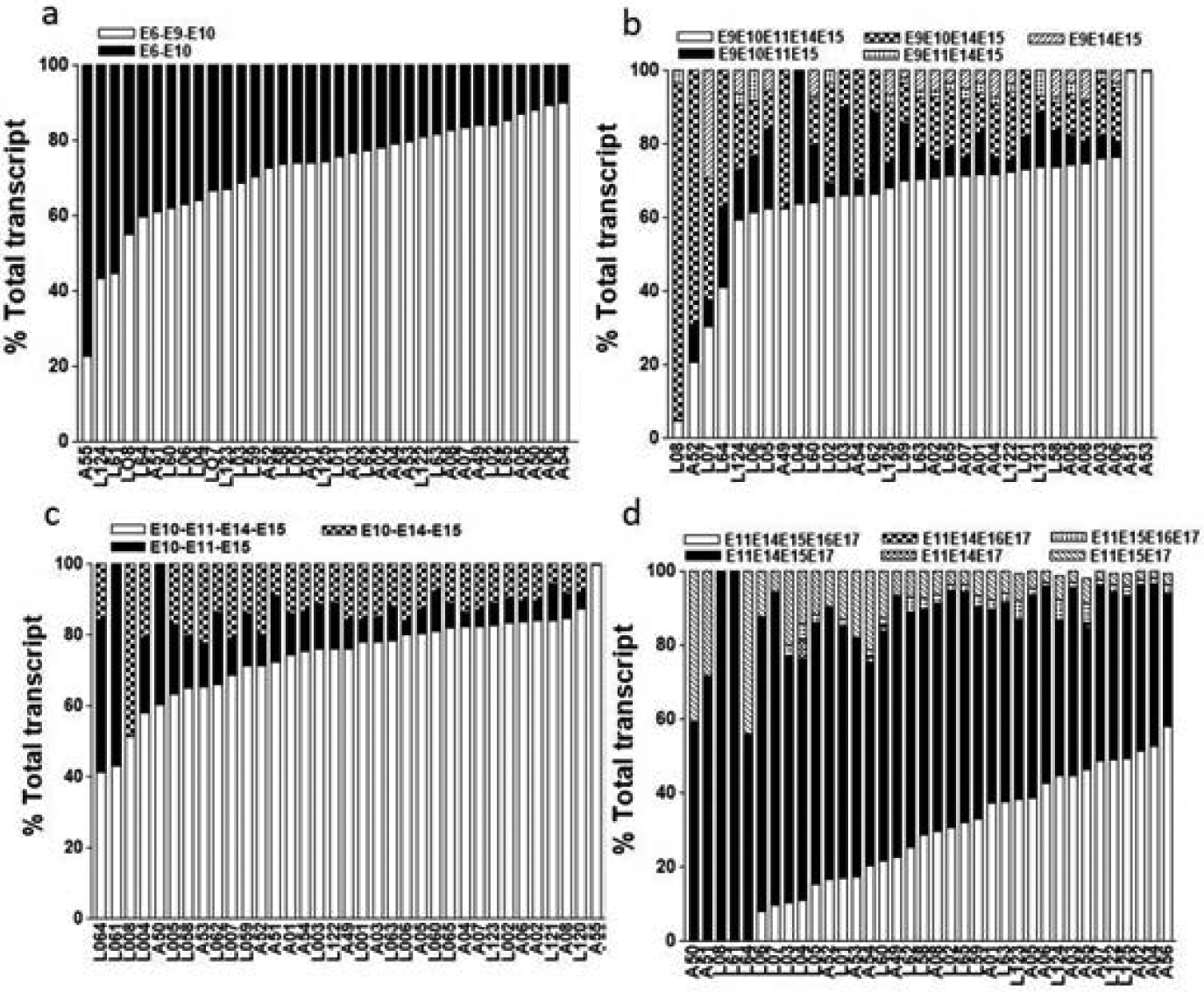
The patterns of ESR1 splice variants initiated from exon 1 (exon 1 to exon 9) (a) or exon 2 (exon 2 to exon 10) (b). The amount of each splice variant was expressed as the percentage of the total transcripts from each locus. Each vertical bar represents the composition of ESR1 splice variants in human liver from different individuals. Each sample was measured twice, and the mean is shown.

The relative expression of splice variants lacking each of six internal coding exons, exon 9, 10,11, 14, 15, and 16, singly or in combination are shown in Table 1 and Figure 5. While proportion of variants lacking exon 10 and 15 or lacking more than one exons are low (<5%), variants lacking exon 9, 11, 14, and 16 comprise a significant portion of total *ESR1* transcripts, even more than the full length transcripts in some livers (e.g. exon 16 skipping variant) (Figure 5d). There is a large inter-person variability in the expression of different splice variants. For example, expression of variant lacking exon 16 is predominant in over 50% of livers tested with some livers only expressing exon 16 skipping variant (Figure 5d).

Interestingly, it appears that the expression levels of some splice variants differ between livers from Caucasian and African American donors. In African American livers, the expression levels of exon 6 containing transcripts initiated from exon 2 (E2E3E6E9E10) is higher (71.4 ± 26% *vs*. 47.5 ± 31%, *p*=0.029) than in Caucasian livers, while the expression of exon 16 skipping variant (E11E14E15E17) is lower (54.5 ± 11.6% *vs*. 64.3 ± 16.4%, *p*=0.044). There are no differences in expression levels of *ESR1* splice variants between age and sex.

### *ESR1* protein expression in human liver

**Figure 6:**
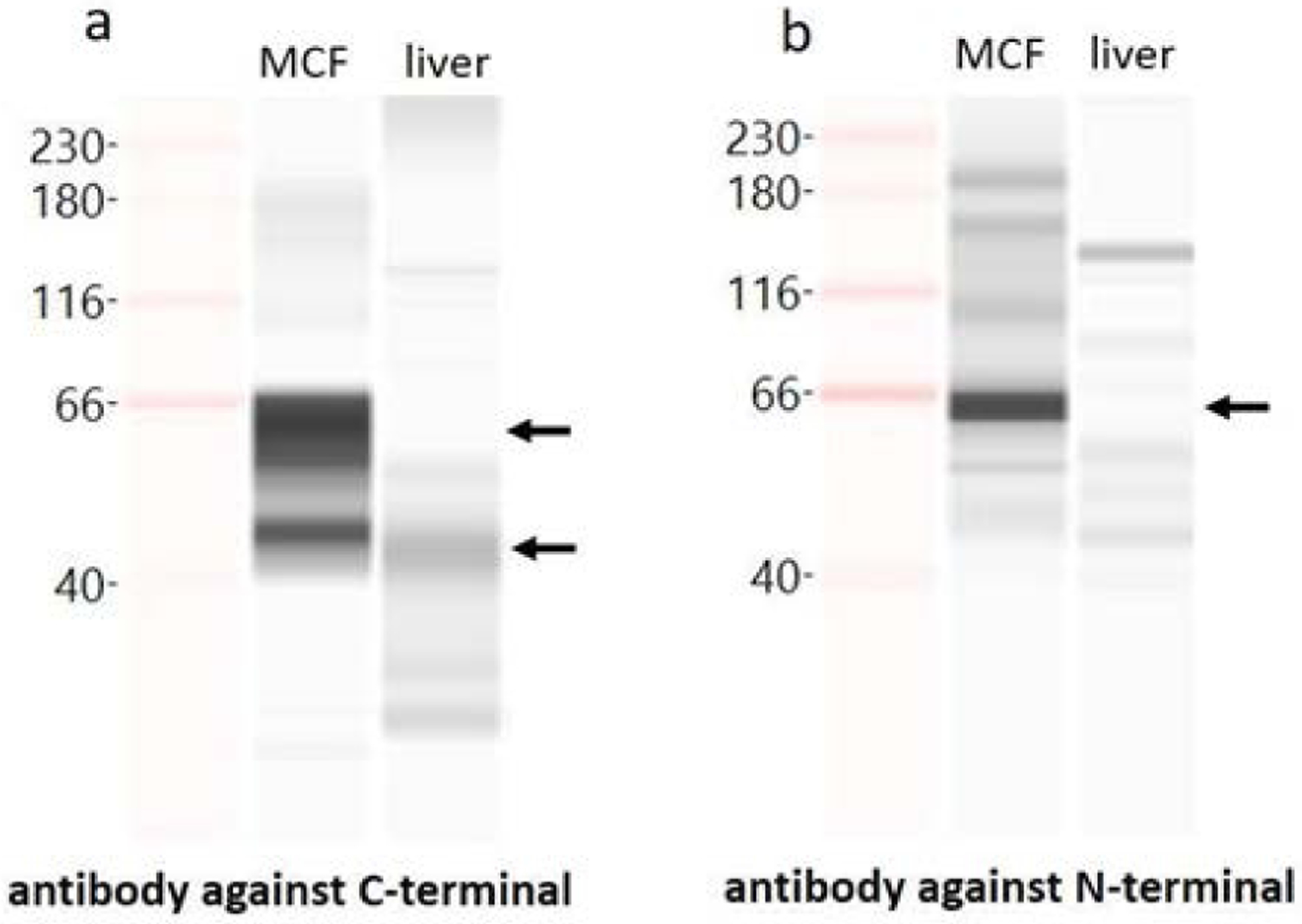
Images of capillary Western Blot with antibodies again C-terminal (a) or N-terminal (b) regions of ESR1, in MCF cells or liver tissue as indicated. Arrows indicate the full-length or N-terminal truncated ESR1 protein at 66 KD or 46 KD, respectively.

When using *ESR1* antibody raised against C-terminal region of *ESR1* protein (clone F-10 antibody, Santa Cruz), Capillary Western Blot analysis showed two bands of *ESR1* protein in MCF7 and liver, corresponding to 66 KD full-length and 46 KD N-terminal truncated *ESR1* protein, respectively (Figure 6a). The major band in MCF7 is at 66 KD, while in liver it is at 46 KD. Whereas, when using *ESR1* antibody raised against N-terminal region of *ESR1* protein (clone D-12 antibody, Santa Cruz), the band at 66 KD is clearly detectable in MCF7 cell but not in liver (Figure 6b). These results indicate that the main isoform of *ESR1* protein in liver is N-terminal truncated isoform, consistent with results from RNA analysis.

## Discussion

In this study, using information from literature and genomic databases (Ensembl, NCBI and GTEx), we re-constructed the *ESR1* genomic organization map. The new version of *ESR1* genomic organization contains 29 unique exons. The expression of these exons was validated either by RNAseq (GTEx) or by real-time PCR previously or in this study. Alternative splicing of *ESR1* exons generates numerous mRNA splice variants either with unique 5’UTRs or encoding *ESR1* proteins lacking functional domains and/or with unique C-terminal structure, in different tissues/cells, including the liver. Employing a quantitative method to measure the expression levels of all known *ESR1* splice variants in 36 human livers, we found extensive inter-individual variations in splicing patterns of *ESR1* transcripts. This study is the first to report the inter-person variability of *ESR1* splicing in human liver. Since different splice variants encode *ESR1* proteins lacking different functional domains and with different trans-activities, the variability in *ESR1* splicing may contribute to variable *ESR1* related gene expression regulation in liver, leading to variable liver functions and the risks of liver diseases.

### Functional consequences of different splice variants

At least six alternative promoters are used by *ESR1* in different tissues. Transcripts starting from exon 6, 5, and 4 differ in their 5’untranslated regions (5’UTR) and splice to a common site 5’ to the translation initiation codon (exon 6c), therefore, generating a common full length *ESR1* protein of 66-kDa (ERα66) [7]. Whereas transcripts starting from exon 1 and 2 undergo further alternative splicing, generating numerous 5’UTR splice variants, some of them with different translation efficiency [11,12] (Supplementary Table 3c). While some of these exon 1 and 2 initiated 5’UTR splice variants do not change the structure of encoded *ESR1* protein, variants that skip first coding exon (exon 6c) encode a shorter *ESR1* protein, denoted as ERα46 that lacks N-terminal 173 amino acids [14]. Moreover, transcripts driven by promoters downstream of exon 6 also encode *ESR1* protein lacking N-terminal domain, for example, *ESR1*-206 or ERα36, which starts from exon 7, lacks exon 16 and 17, and uses an alternative terminal exon, exon 18 [22]. It is unclear whether exon EX8, EX9, EX15, or EX16 reported in NCBI database (Figure S1b) are first exons or merely an incomplete cDNA sequence.

Our results showed liver *ESR1* mRNA is mainly initiated from exon 1, followed by exon 2 and 6, consistent with a previous study [26] (Figure 3b). The majority of exon 1 initiated transcripts, for example, E1E3E9 and E1E9, skip exon 6, indicating N-terminal truncated isoform is a main *ESR1* isoform in the liver (Figure 4a). This result is supported by capillary western blot analysis showing N-terminal truncated 46 KD isoform is the major isoform in liver (Figure 6). While the majority of exon 2 initiated transcripts contain exon 6 (for example, E2E3E6E9E10), there is a large inter-person variability in relative expression levels, ranging from 0–100% of total transcripts in 36 human livers, indicating highly variable expression of *ESR1* transcripts containing exon 6 in human livers (Figure 4b). N-terminal truncated *ESR1* isoform, like ERα46, missing N-terminal AF1 domain, exhibits either ligand-inducible transactivation or dominant negative effects on ERα66, depending on the cellular context [14]. Moreover, the ratio of ERα46/ERα66 changes with the cell growth status of the breast carcinoma cell line MCF7 [14]. Since hepatocellular carcinoma derived HepG2 cells appear to mediate *ESR1* signalling through the AF-1 transactivation function, *ESR1* with N-terminal truncation may have dominant negative effect in liver, regulating the trans-activity of ERα66 [27,28]. However, we cannot rule out the possibility that ERα46 may have unique function in liver, which requires further investigation.

*ESR1* splice variants with internal exon deletion or insertion of ‘intronic’ exons generate numerous C-terminal truncated *ESR1* proteins [13]. Although more than one exon deletion or insertion of ‘intronic’ exons is rare, deletion of each of six internal coding exon is readily detectable in liver (Figure 3d and Figure 5). Again, there is a large inter-person variability in the expression of these exon-skipping transcripts, with some individuals only express exon-skipping variants (for example, ΔE16 in Figure 5d). Skipping of exon 10 or 11 is in frame deletion, missing 39 and 112 amino acids in DNA binding or hinge domain, respectively. Whereas deletion of exon 9, 14, 15, or 16 shifts open reading frame, encoding C-terminal truncated *ESR1* proteins with adding 4 to 60 unique amino acid at C-terminal end (Supplementary Table 3d for protein structure changes of these variants. Previous cell transfection studies showed variants with deletion each of six internal coding exons encode stable *ESR1* proteins with expected molecular weight, displaying different DNA binding, subcellular distribution, ligand binding and transcriptional activity [29]. For example, variant with exon 10 (third coding exon) deletion has normal ligand binding activity and nucleus localization, but completely loss DNA binding activity to a consensus estrogen responsive element (ERE). However, this variant remains binding activity to steroid receptor coactivator-1e (SRC-1e) and exert transcription activity with ovalbumin promoter, which contains an ERE half-site and an AP-1 motif, in a ligand dependent fashion [29]. Variant with exon 14 (5th coding exon) deletion has normal nucleus localization, but reduced DNA binding activity to a consensus ERE and completely loss estradiol binding activity. In transfected cells, this variant exhibited constitutive transactivation of an ERE-driven promoter in the absence of estrogen [13]. Although exon 10 skipping is a rare event, exon 14 skipping is relatively frequent in liver, with large inter-person variability, ranging from 0–66% of total transcripts in different individuals. Individuals with higher level of exon 14 skipping variants of *ESR1* may have different liver gene expression regulatory networks compared to individuals expressing normal *ESR1* transcripts. *ESR1* variants with exon 9, 11, 15, or 16 deletions have impaired DNA-binding, ligand binding, and nuclear localization capability, leading to *ESR1* proteins without transcriptional activity [29]. Livers with higher expression levels of these variants are expected to have reduced *ESR1* activity in general. However, we cannot rule out the possibility that these C-terminal truncated *ESR1* variants have other functions, for example, non-genomic estrogen signaling as reported for ERα36, binding to DNA motif other than ERE motif and more [30,31]. Moreover, although *ESR1* is considered as a ligand-activated DNA binding transcription factor, binding of the unliganded form of *ESR1* to promoters of target genes has been reported recently by ChIPseq assay [1]. We have recently identified ligand-free *ESR1* as a master regulator for the expression of CYP3A4 and other cytochrome P450s in liver (in press). Since ligand-binding domain of *ESR1* is located at C-terminal and many exon-skipping *ESR1* variants encoding C-terminal truncated *ESR1* protein, some exhibit constitutive activity, for example, variant with exon 14 skipping. It is plausible to consider that the ligand-free activity of *ESR1* in liver may be mediated by these C-terminal truncated *ESR1* isoforms.

### Causes of variable expression of *ESR1* splice variants

Alternative splicing is regulated by multiple factors, acting through both cis-acting and trans-acting pathways [32]. cis-acting elements include the DNA sequences required for efficient splicing, that is, 5’ splice site, 3’ splice site, branch sites and Py tract, as well as intronic or exonic splicing enhancer and silencer. Early study demonstrated that an intronic SNP rs2273207 was associated with *ESR1* splice variant missing exon 16, with G allele associating with higher level of *ESR1* splice variant missing exon 16. However, our study cohort is too small to allow genetic association study, but we did observe a racial difference in the expression of variant missing exon 16, with livers from Caucasian American donor having higher level of variant missing exon 16. Since rs2273207 G allele in European decedents is much more frequent than in African decedents (0.89 *vs*. 0.53), it is possible that the racial difference in variants with exon 16 skipping may be driven by different allele frequency of rs2273207 in these two groups. This need to be tested in a larger cohort. Moreover, we also observed a racial different in the expression of exon 2 initiated transcripts containing exon 6, with African American livers having higher level of exon 6 containing variants than Caucasian livers. Whether the difference is caused by genetic or non-genetic factors requires further investigation.

It is worth of noting that relative expression levels of *ESR1* splice variants obtained from GTEx RNAseq results are drastically different from our results measured with real-time PCR. While GTEx data show *ESR1*-210 and *ESR1*-206, initiating from exon 4b and exon 7, respectively, are the main transcripts in most of the tissues/cells analyzed including breast, liver, heart, lung, etc., our real-time PCR result indicates low expression level of exon 4 and 7 in these tissues (Figure 2 and Figure 3b). Instead, the main initiating exons in these tissues are exons 1, 2 and 6 (Figure 3b). Since real-time PCR is considered as a gold standard method for gene expression, these results indicate the limitation of short-read RNAseq technology to accurately quantify the relative expression levels of different transcripts in complex gene locus like *ESR1*. However, real-time PCR or PCR with fluorescently labeled primer methods can only provide information of exon usage at specific splice locus without information of whole transcript. Future studies will focus on using long-read RNAseq technology, for example, PacBio SMRT or Nanopore sequencing, to accurately quantitate the expression of *ESR1* splice variants at whole transcript level.

## Conclusion

In summary, the results presented here revealed a large degree of inter-individual variability in *ESR1* mRNA splice variants, likely to mediate substantial phenotypic variation of *ESR1*. Since most of the splice variants do encode stable protein and may exert different degree of activities, from dominant negative effects, no activity, normal activity to constitutive activity, the inter-person variability in the composition of *ESR1* transcripts is likely to play a role in diverse liver gene expression regulation, drug metabolism and liver diseases. Future studies will focus on understanding the function of different splice variants in liver and identifying genetic or other factors that contribute to variable *ESR1* splicing.

## Supplementary Material

1

## Figures and Tables

**Table 1:** Quantitative analysis of ESR1 splice variants in human liver (n=36) using PCR with fluorescently labeled primers.

% Total transcripts inside the locus						
Locus	Splice Variants	Mean ± SD	Range (min to max)	Amplicon size (bp)	Changes in mRNA structure	Changes in protein structure
E1 to E9	E1E9	17 **±** 13	0 ~ 55	98	ΔE3 + ΔE6	N-terminal truncation
	E1E3E9	79 **±** 14	45 ~ 100	229	ΔE6	
	E1N2E3E9	2 **±** 2	0 ~ 6	288	ΔE6, insert N2	
	E1E6E9	0.3 **±** 0.7	0 ~ 4	620	ΔE3	No change
	E1E3E6E9	3 **±** 6	0 ~ 22	751	Reference	Reference
E2 to E10	E2E9E10	3 **±** 7	0 ~ 25	282	ΔE3 + ΔE6	N-terminal truncation
	E2E3E9E10	31 **±** 31	0 ~ 100	413	ΔE6	
	E2E3E6E10	7 **±** 6	0 ~ 20	744	ΔE9	C-terminal truncation
	E2E6E9E10	0.6 **±** 1.2	0 ~ 4	804	ΔE3	No change
	E2E3E6E9E10	58 **±** 31 **±** 31	0 ~ 100 0 ~ 100	935	Reference	Reference
E6 to E10	E6E10	28 **±** 14	9 ~ 77	124	ΔE9	C-terminal truncation
	E6E9E10	72 **±** 14	23 ~ 90	315	Reference	Reference
E9 to E15	E9E14E15	3 **±** 5	0 ~ 30	394	ΔE10 + ΔE11	C-terminal truncation
	E9E10E14E15	19 **±** 19	0~ 91	511	ΔE11	
	E9E10E11E15	10 **±** 8	0 ~ 36	709	ΔE14	
	E9E11E14E15	2 **±** 2	0 ~ 8	731	ΔE10	
	E9E10E11E14E15	66 **±** 19	5 ~ 100	848	Reference	Reference
E10 to E15	E10E14E15	13 **±** 8	0 ~ 48	282	ΔE11	C-terminal truncation
	E10E11E15	12 **±** 12	0 ~ 56	480	ΔE14	
	E10E11E14E15	75 **±** 13	42 ~ 100	619	Reference	Reference
E11 to E17	E11E15E17	10 **±** 10	0 ~ 44	211	ΔE14 + ΔE16	--
	E11E14E17	0.4 **±** 1	0 ~ 6	216	ΔE15 + ΔE16	C-terminal truncation
	E11E14E15E17	60 **±** 15	35 ~ 100	350	ΔE16	
	E11E15E16E17	2 **±** 2	0 ~ 6	395	ΔE14	
	E11E14E16E17	0.1 **±** 0.1	0 ~ 0.6	400	ΔE15	--
	E11i45asE15E16E17	0.1 **±** 0.2	0 ~ 0.7	503	ΔE14+inserti45 as	--
	E11E14E15E16E17	28 **±** 18	0 ~ 58	534	Reference	Reference
