# Supplementary material for: Highly Variable Expression of ESR1 Splice Variants in Human Liver: Implication in the Liver Gene Expression Regulation and Inter-Person Variability in Drug Metabolism and Liver Related Diseases
Source: J Mol Genet Med. Author manuscript; Available in PMC 2020 May 26. (PMC7249510)
Supplement: 1 [file NIHMS1067622-supplement-1.pdf]

Table S1. Demographics of liver donors

Sample ID	Race	Sex	Age
L001	W	F	49
L002	W	F	14
L003	W	M	74
L004	W	F	53
L005	W	F	63
L006	W	M	51
L007	W	M	53
L008	W	M	49
L058	W	F	48
L059	U	M	37
L060	W	M	79
L061	U	F	77
L062	W	M	63
L063	W	F	75
L064	W	M	66
L065	W	M	54
L121	W	M	68
L122	W	F	65
L124	W	M	65
L125	W	F	77
A01	B	F	64
A02	B	F	59
A03	B	F	69
A04	B	F	60
A05	B	F	52
A06	B	M	62
A07	B	M	54
A08	B	M	44
A49	B	F	71
A50	B	F	53
A51	B	F	58
A52	B	F	78
A53	B	F	78
A54	B	M	18
A55	B	M	78
A56	B	M	83

Table S2 Sequences of PCR primers

	Sequence	
**Real-time PCR**		
ESR1 total expression	F: AATCTGCAGGGAGAGGAGTTTGT	
	R: ACTCGGTGGATATGGTCCTTCTC	
ESR1 exon 1	F: ATCCTTTTGATTGTGAATTATATTCTGTAGC	
	R: GAGATCTTTGTGCTTACTCCTTCCTAGT	
ESR1 exon 2	F: AGAAGACAGTCTCTGAGTGATAATCTTCTCT	
	R: GAGATCTTTGTGCTTACTCCTTCCTAGT	
ESR1 exon 4	F: CTGGAAAGACGTTCTTGATCCAG	
	R: GGGCAGAAGGCTCAGAAACC	
ESR1 exon 5	F: GGCCGTGAAACTCAGCCTC	
	R: CGGGCCACTTTACTTGTCGT	
ESR1 Exon 6	F: GCCCGCCGGCATTC	
	R: TCTGCCACCCTGGCGT	
ESR1 exon 7	F: CCAGGACACAGGAGACCATTTGTG	
	R: TCGATTATCTGAATTTGGCCTTC	
ESR1 exon X3	F: CCACCCAAGGAATTCAGGTTC	
	R: GAGATCTTTGTGCTTACTCCTTCCTAGT	
ESR1 exon X5	F: CTCAGCTTTGTAAAATAATGTAATCAAGGA	
	R: GAGATCTTTGTGCTTACTCCTTCCTAGT	
ESR1 exon X1	F: AGAACCTTGGATCCTGACCTCA	
	R: GGGCAGAAGGCTCAGAAACC	
ESR1 exon X9	F: TCTTATGGAGACATGTGGCTTTCA	
	R: GGGCAGAAGGCTCAGAAACC	
ESR1 exon X8	F: GAGGATTGCTGTTCTCCATGG	
	R: CCTTGTCATTGGTACTGGCCA	
ESR1 exon 4L	F: ATCAACTGGGCGAAGAGGG	
	R: TTAAAAGCTGCGCTTCGCAT	
ESR1 exon i45a	F: ATCAACTGGGCGAAGAGGG	
	R: TGTTCTTTTCCATCCTGAGAGATTG	
ESR1 exon i45b	F: ATCAACTGGGCGAAGAGGG	
	R: CCTGGAGGAGAGGAACCTGG	
ESR1 exon i45c	F: ATCAACTGGGCGAAGAGGG	
	R: CACCCAGGATCACACACATATCC	
ESR1 exon i56	F: CCTAACTTGCTCTTGGACAGGATC	
	R: CACTGCGCTCCTGTCTAGGTG	
ESR1 exon i67	F: GGGAGAGGAGTTTGTGTGCCT	
	R: ATGGGCGTGTGACCCACT	
**PCR with fluorescently labeled primers**		
E1-E9 locus	F: ATCCTTTTGATTGTGAATTATATTCTGTAGC	FAM
	R: CCTTGTCATTGGTACTGGCCA	
E2-E10 locus	F: AGAAGACAGTCTCTGAGTGATAATCTTCTCT	
	R: CCTCCTGTTTTTATCAATGGTGC	FAM
E6-E10 locus	F: CTACTACCTGGAGAACGAGCCCAG	

	R: CCTCCTGTTTTTATCAATGGTGC	FAM
E9-E15 locus	F: ATAATCGACGCCAGGGTGG	
	R: GATCTCTAGCCAGGCACATTCTAGA	FAM
E10-E15 locus	F: ATTGATAAAAACAGGAGGAAGAGCTG	
	R: GATCTCTAGCCAGGCACATTCTAGA	FAM
E11-E17 locus	F: CGATGATGGGCTTACTGACCA	
	R: GCACCACGTTCTTGCACT	FAM
**Primers used for cDNA synthesis**		
	ATCAGGTGGATCAAAGTGTCTGTG	
	GGGATCTTGAGCTGCGGA	
	GCAAATGAGAAAACTGAAGGCC	
	GGGTAAAATGCAGCAGGGATTA	
	AGGCCTTGCAGCCCTCA	
	TTCTCCCATGACATCACAACAAG	
	GAGATCATTCTGGCAGTAGAGTAGTTTTAA	
	CACCTGGAAGTTGCAATAGAAAGAC	
	GGTGACAAGCTGGAAATCTAAGCT	
	CAGGCATGATGGCGCAT	

Table S3. ESR1 transcripts and splice variants reported in both databases and in the literature

Table S3a. ESR1 transcripts reported in Ensembl database

Isoform name	Transcript ID	Length (bp)	Biotype	Protein size
ESR1-201	ENST00000206249.7	6458	protein coding	595aa
ESR1-202	ENST00000338799.9	3335	protein coding	595aa
ESR1-203	ENST00000404742.5	624	protein coding	84aa
ESR1-204	ENST00000406599.5	1251	protein coding	334aa
ESR1-205	ENST00000415488.1	323	protein coding	107aa
ESR1-206	ENST00000427531.6	5436	protein coding	310aa
ESR1-207	ENST00000440973.5	6466	protein coding	595aa
ESR1-208	ENST00000443427.5	6357	protein coding	595aa
ESR1-209	ENST00000446550.1	731	protein coding	115aa
ESR1-210	ENST00000456483.3	1368	protein coding	152aa
ESR1-211	ENST00000473497.5	219	processed transcript	no protein
ESR1-212	ENST00000482101.1	641	processed transcript	no protein
ESR1-213	ENST00000488573.1	530	processed transcript	no protein
ESR1-214	ENST00000638569.1	132	protein coding	44aa
ESR1-215	ENST00000641399.1	1207	processed transcript	no protein

Table S3b. ESR1 transcripts reported in NCBI database

Isoform name	Transcript ID	Length (bp)	Biotype	Protein size	Protein ID	Distinct exon
Variant 1	NM_000125.3	6330	protein coding	595 aa	NP_000116.2	E6b
Variant 2	NM_001122740.1	6357	protein coding	595 aa	NP_001116212.1	same as ESR1-208
Variant 3	NM_001122741.1	6314	protein coding	595 aa	NP_001116213.1	E4b
Variant 4	NM_001122742.1	6466	protein coding	595 aa	NP_001116214.1	same as ESR1-207
Variant 5[a]	NM_001291230.1	6320	protein coding	595 aa	NP_001278159.1	E4b, splice variant
Variant 6[a]	NM_001291241.1	6311	protein coding	594 aa	NP_001278170.1	E4b, polymorphism
Variant X1	XM_011535543.2	5769	protein coding	595 aa	XP_011533845.1	EX1 or E3
Variant X2	XM_017010376.1	6391	protein coding	595 aa	XP_016865865.1	5' splice variant
Variant X3	XM_017010377.1	6450	protein coding	595 aa	XP_016865866.1	EX3 or N2
Variant X4	XM_017010378.1	3902	protein coding	595 aa	XP_016865867.1	short E17
Variant X5	XM_017010379.1	3936	protein coding	595 aa	XP_016865868.1	EX5
Variant X6	XM_017010380.1	6260	protein coding	595 aa	XP_016865869.1	5' splice variant
Variant X7	XM_011535544.2	3953	protein coding	595 aa	XP_011533846.1	E5a
Variant X8	XM_017010381.1	3688	protein coding	595 aa	XP_016865870.1	EX8
Variant X9	XM_011535545.2	3842	protein coding	595 aa	XP_011533847.1	EX9 or T2
Variant X10	XM_011535547.2	2888	protein coding	483 aa	XP_011533849.1	i67
Variant X11	XM_006715374.3	6282	protein coding	466 aa	XP_006715437.1	splice variant
Variant 12	XM_006715375.3	3941	protein coding	422 aa	XP_006715438.1	partial I6 retention
Variant X15	XM_011535549.2	2955	protein coding	352 aa	XP_011533851.1	EX15
Variant X16	XM_017010383.1	3313	protein coding	332 aa	XP_016865872.1	EX16
Variant X17	XM_017010382.2	3058	protein coding	376 aa	XP_01686587.1	splice variant

[a.] compared to variant 3, variant 5 has a 6bp insertion in exon 10, leading to two aa (Asn-Ser) insertion after Gly212, while variant 6 has a 3bp deletion in exon 10 at different location, leading to a Gly253 deletion

Table S3c. Known 5'UTR splice variants of transcripts initiated from exon 1 and exon 2.

5' end mRNA sequence	Change in mRNA	Change in protein
**Initiated from E1**		
E1-E6	shorter 5'UTR	no change
E1-E3-E6	reference sequence	no change
E1-N1-E3-E6	longer 5'UTR	no change
E1-N2-E3-E6	longer 5'UTR	no change
E1-E9	shorter 5'UTR and ΔE6	N-terminal truncation
E1-E3-E9	ΔE6	N-terminal truncation
E1-N2-E3-E9	longer 5'UTR and ΔE6	N-terminal truncation
**Initiated from E2**		
E2-E6	shorter 5'UTR	no change
E2-E3-E6	reference sequence	no change
E2-E3-X1-E6	longer 5'UTR	no change
E2-E9	shorter 5'UTR and ΔE6	N-terminal truncation
E2-E3-E9	ΔE6	N-terminal truncation
E2-E3-X1-E9	longer 5'UTR and ΔE6	N-terminal truncation

Table S3d. Splice variants with internal exon deletion

Exon deletion	Transcript structure	Protein size (aa)	Protein structure
ΔE9	E6-E10-E11-E14-E15-E16-E17	151	C-terminal truncation and unique last aa
ΔE10	E6-E9-E11-E14-E15-E16-E17	556	In frame deletion, missing 39 aa, lacking DNA binding domain
ΔE11	E6-E9-E10-E14-E15-E16-E17	483	In frame deletion, missing 112 aa, lacking hinge and ligand-binding domains and partial DNA binding domain
ΔE14	E6-E9-E10-E15-E16-E17	371	C-terminal truncation and unique amino acid sequence for the last 6 aa
ΔE15	E6-E9-E10-E11-E14-E16-E17	470	C-terminal truncation and unique amino acid sequence for the last 60 aa
Δ16	E6-E9-E10-E11-E15-E16-E17	450	C-terminal truncation and unique amino acid sequence for the last 10 aa

Table S3e. Splice variants with intronic exon insertion*

Transcript Name	Transcript structure	Protein size (aa)	Protein structure
CTERP-1	E6-E9-E10-E11i11 (4L)	373	C-terminal truncation and unique amino acid sequence for the last 8 aa
ERai45aL	E6-E9-E10-E11-i45aL	386	C-terminal truncation and unique amino acid sequence for the last 21 aa
ERai45aS	E6-E9-E10-E11-i45aS-E14-E15-E16-E17	386	C-terminal truncation and unique amino acid sequence for the last 21 aa
ERai45bL	E6-E9-E10-E11-i45bL	452	C-terminal truncation and unique amino acid sequence for the last 87 aa
ERa45bS	E6-E9-E10-E11-i45bS	400	C-terminal truncation and unique amino acid sequence for the last 35 aa
ERai45c	E6-E9-E10-E11-i45c-E14-E15-E16-E17	382	C-terminal truncation and unique amino acid sequence for the last 17 aa
ERai56	E6-E9-E10-E11-E14-i56	424	C-terminal truncation and unique amino acid sequence for the last 13 aa
ERaDup5	E6-E9-E10-E11-E14-E14-E15-E16-E17	419	C-terminal truncation and unique amino acid sequence for the last 8 aa
ERai67	E6-E9-E10-E11-E14-E15-i67	483	C-terminal truncation and unique amino acid sequence for the last 27 aa

* reference #13

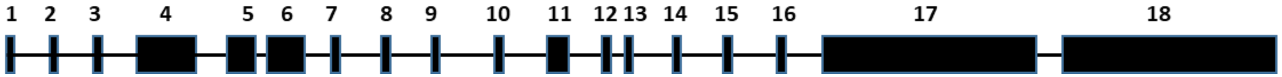

a.

ESR1-201 E6a---------------E9----E10---E11----------E14--E15--E16---E17L
ESR1-202 E4a--------- E6c-------------- E9----E10---E11---------E14--E15--E16---E17S
ESR1-203 E2--E3------------------E6c
ESR1-204 E6b---E15--E16---E17S
ESR1-205 E10-----------------E14--E15
ESR1-206 E7----------E9----E10---E11----------E14--E15--E18
ESR1-207 E2—E3-----------------E6c----------------E9----E10---E11---------E14--E15--E16---E17L
ESR1-208 E5b-E6c----------------E9----E10---E11----------E14--E15--E16---E17L
ESR1-209 E5a-E6c
ESR1-210 E4b----------E6c----------------E9----E10----------------E14--E15---E16----E17L
ESR1-211 E1--------E3------------------E6c
ESR1-212 E11-E12-E13
ESR1-213 E6--------E8

b.

Variant 1 E6b-------------- E9----E10----E11--------E14---E15---E16----E17L
Variant 2 E5b-E6c---------------E9----E10----E11--------E14---E15---E16----E17L
Variant 3,5,6 E4b----------E6c---------------E9----E10----E11--------E14---E15---E16----E17L
Variant 4,X4 E2--E3-----------------E6c---------------E9----E10----E11--------E14---E15---E16----E17L
Variant X1 E2--E3—EX1---------E6c---------------E9----E10----E11--------E14---E15---E16----E17
Variant X2 E1--------E3-----------------E6c---------------E9----E10----E11--------E14---E15---E16----E17
Variant X3 E1-EX3--E3-----------------E6c---------------E9----E10----E11--------E14---E15---E16----E17
Variant X5 EX5-E3-----------------E6c---------------E9----E10----E11--------E14---E15---E16----E17
Variant X6 E1---------------------------E6c--------------E9----E10----E11--------E14---E15---E16----E17
Variant X7 E5a-E6c--------------E9----E10----E11--------E14---E15---E16----E17L
Variant X8 EX8--E9----E10----E11--------E14----E15---E16---E17
Variant X9 EX9----E6c--------------E9----E10----E11--------E14----E15---E16---E17
Variant X10 E2--E3---------------E6c---------------E9----E10----E11--------E14----E15-i67
Variant X11 E2--E3---------------E6c---------------E9----E10----E11--------E14----E15---E18
Variant X12 E6a-I6-----------E9----E10----E11--------E14----E15---E16---E17L
Variant X15 EX15----E11--------E14----E15---E16---E17
Variant X16 EX16----E11--------E14----E15----E16---E17
Variant X17 E6c---------------------E10----E11--------E14----E15----E16---E17

Figure S1. The structure of ESR1 transcripts reported in Ensembl (a) and NCBI (b) databases.

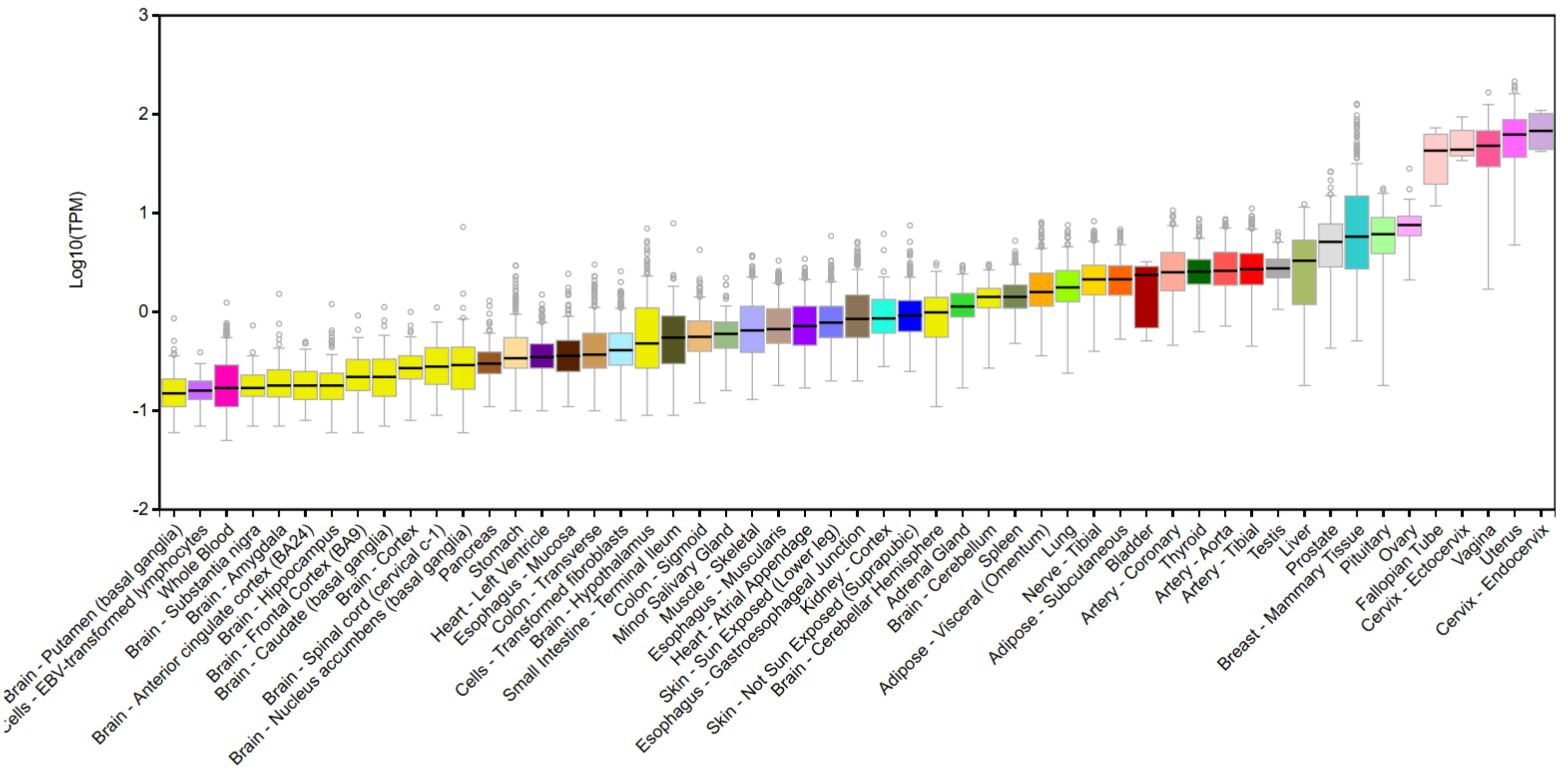

Figure S2. ESR1 expression levels in different tissues measured using RNAseq (data from GTEx).

a

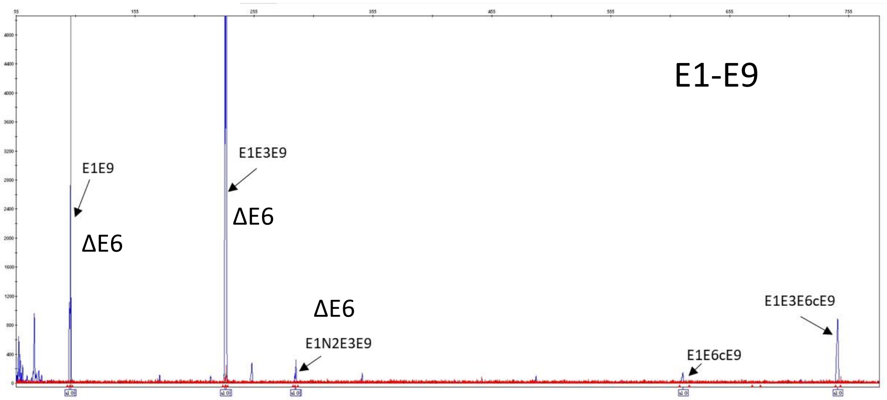

b

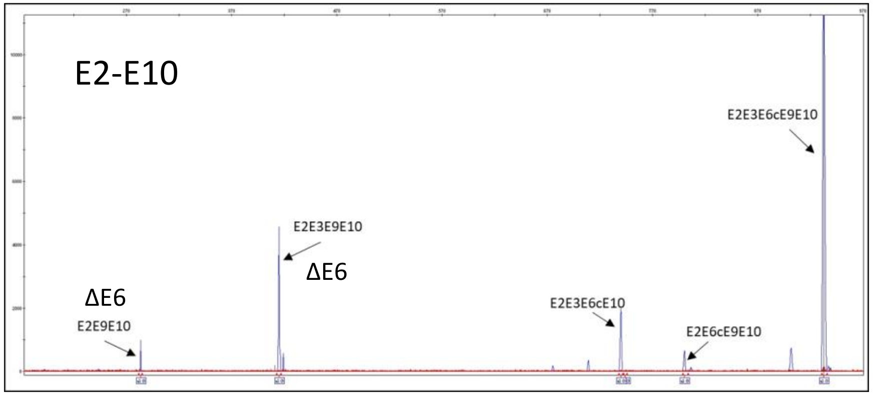

Figure S3. Detection of splice variants initiated from exon 1 (a) or exon 2 (b). Complementary DNA fragments spanning exon 1 to exon 9 or exon 2 to exon 10 were amplified with a pair of primers shown in Table S2, with one of which labeled with FAM fluorescence dye. The PCR products were run on SeqStudio capillary electrophoresis DNA analyzer. The differently sized PCR products were separated, and the splice variants were identified by their sizes.

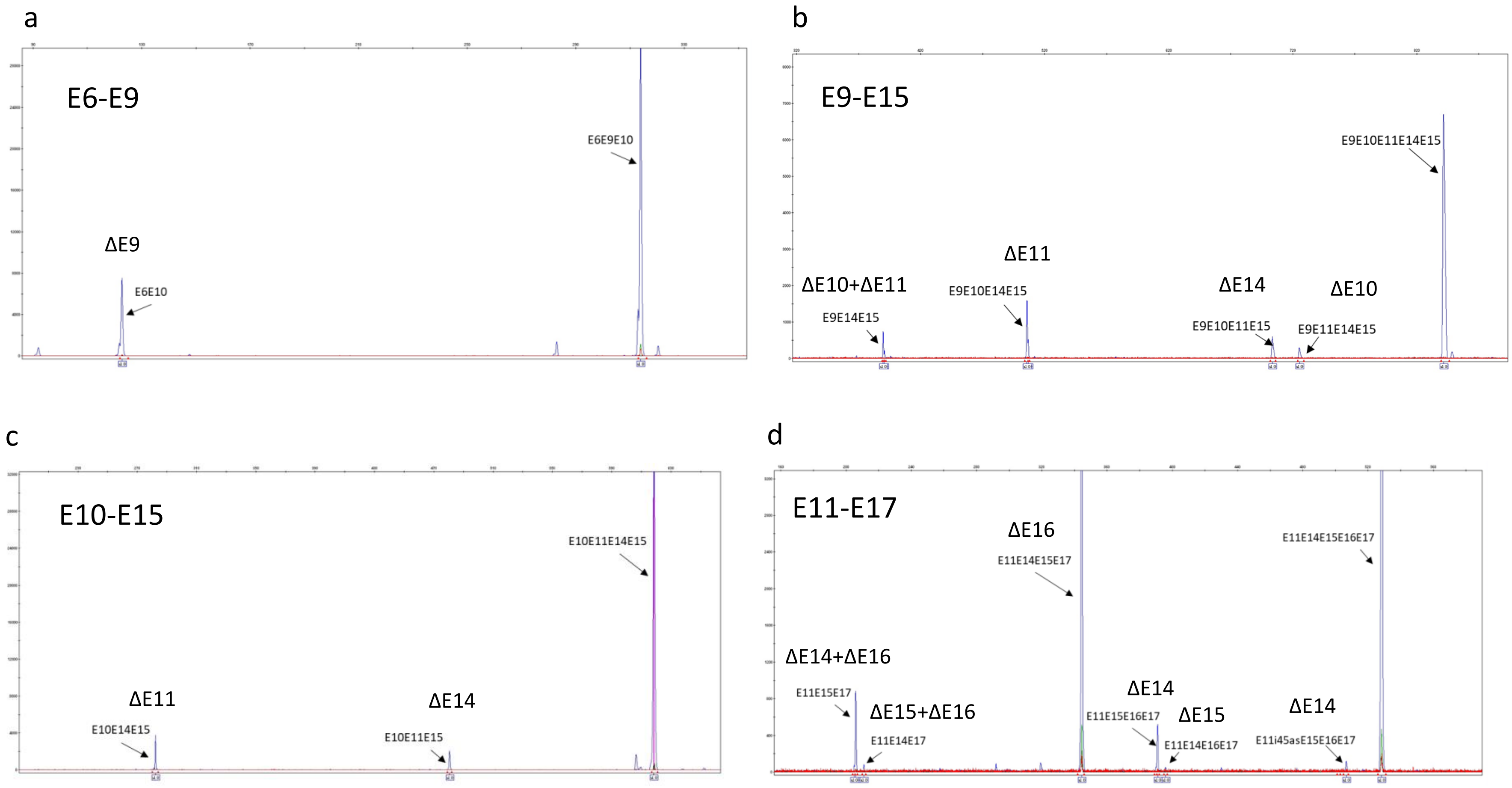

Figure S4. Detection of splice variants derived from loci E6-E9 (a), E9-E15 (b), E10-E15 (c) or E11-E17 (d). Complementary DNA fragments spanning different loci were amplified with a pair of primers shown in Table S2, with one of which labeled with FAM fluorescence dye. The PCR products were run on SeqStudio capillary electrophoresis DNA analyzer. The differently sized PCR products were separated, and the splice variants were identified by their sizes.
